# Analysis of Serial Multidrug-Resistant Tuberculosis Strains Causing Treatment Failure and Within-Host Evolution by Whole-Genome Sequencing

**DOI:** 10.1128/mSphere.00884-20

**Published:** 2020-12-23

**Authors:** Xinchang Chen, Guiqing He, Siran Lin, Shiyong Wang, Feng Sun, Jiazhen Chen, Wenhong Zhang

**Affiliations:** a Department of Infectious Diseases, Huashan Hospital, Fudan University, Shanghai, China; b Department of Infectious Diseases, Wenzhou Central Hospital, Affiliated Dingli Clinical Institute of Wenzhou Medical University, Wenzhou, China; c State Key Laboratory of Genetic Engineering, School of Life Science, Fudan University, Shanghai, China; d National Clinical Research Center for Aging and Medicine, Huashan Hospital, Fudan University, Shanghai, China; e Key Laboratory of Medical Molecular Virology (MOE/MOH) and Institutes of Biomedical Sciences, Shanghai Medical College, Fudan University, Shanghai, China; University of Nebraska Medical Center

**Keywords:** drug resistance, whole-genome sequencing, multidrug-resistant tuberculosis, within-host evolution, treatment outcome, DNA sequencing, *Mycobacterium tuberculosis*, adaptive resistance, drug resistance evolution, genome analysis, heteroresistance, multidrug resistance

## Abstract

The cure rate of multidrug-resistant tuberculosis (MDR-TB) is relatively low in China. The reasons for the treatment failure and within-host evolution during treatment have not been sufficiently studied. All MDR-TB patients receiving standard treatment from January 2014 to September 2016 at a designated TB Hospital in Zhejiang Province were retrospectively included and grouped according to their known treatment outcome. Clinical information was collected. Baseline strains of all patients and serial strains of treatment-failure patients were revived. Drug susceptibility tests (DSTs) of 14 drugs and single nucleotide polymorphism (SNP) analysis based on whole-genome sequencing (WGS) were performed. The genetic distance and within-host evolution were investigated based on SNPs. In total, 20 treatment failure patients and 74 patients who succeeded in treatment were included. The number of effective drugs for patients who failed treatment was no more than three. Eighteen (90.0%) treatment-failure patients were characterized by a continuous infection of the primary strain, of which 14 patients (77.8%) developed phenotypic or genotypic acquired drug resistance under ineffective treatment. Acquired resistance to amikacin and moxifloxacin (2.0 mg/ml) was detected most frequently, in 5 and 4 patients, respectively. The insufficient number of effective drugs in the combined treatment regimen was the main reason for MDR-TB treatment failure. The study emphasizes the importance of DST for second-line drugs when implementing the second-line drug regimen in MDR-TB patients. For patients with risk factors for MDR-TB, DST of second-line antituberculosis drugs should be performed at initiation of treatment. Second-line drugs should be selected based on the results of DST to avoid acquired resistance. WGS detects low-frequency resistance mutations and heterogeneous resistance with high sensitivity, which is of great significance for guiding clinical treatment and preventing acquired resistance.

**IMPORTANCE** Few studies have focused on the reasons for the low cure rate of multidrug-resistant tuberculosis in China and within-host evolution during treatment, which is of great significance for improving clinical treatment regimens. Acquired resistance events were common during the ineffective treatment, among which resistance to amikacin and high-level moxifloxacin were the most common. The main reason for the treatment failure of MDR-TB patients was insufficient effective drugs, which may lead to higher levels of drug resistance in MDR-TB strains. Therefore, the study emphasizes the importance of DST in the development of second-line treatment regimen when there is a risk of MDR. By performing whole-genome sequencing of serial strains from patients with treatment failure, we found that WGS can detect low-frequency resistance mutations and heterogeneous resistance with high sensitivity. It is thus recommended to conduct drug susceptibility tests at the beginning of treatment and repeat the DST when the sputum bacteria remain positive.

## Abstract (precis)

Few studies have focused on the reasons for the low cure rate of multidrug-resistant tuberculosis in China and within-host evolution during treatment, which is of great significance for improving clinical treatment regimens. Acquired resistance events were common during the ineffective treatment, among which resistance to amikacin and high-level moxifloxacin were the most common.

## INTRODUCTION

Drug-resistant tuberculosis (DR-TB) constitutes one of the main threats to the End-TB by 2035 strategy of the World Health Organization (WHO) (1–3). China accounts for 13% of the worldwide cases of multidrug resistant (MDR)/resistant to rifampin (RR)-TB. However, the cure rate of MDR-TB in China is as low as 41% (3). Risk factors for treatment failure include health and socioeconomic characteristics of patients, such as homelessness and anemia, as well as bacterial factors, such as infection by lineage 2 and higher resistance strains (4–11). The success rate of treatment is positively correlated with the number of effective drugs administered to patients (12, 13).

All MDR-TB patients who failed standardized treatment in accordance with the 2011 version of the WHO guidelines (14) were retrospectively analyzed in a designated TB Hospital in Zhejiang Province, China from January 2014 to September 2016 to investigate the current status and reasons for MDR-TB treatment failure. Due to the high occurrence and transmission rate of MDR-TB in China (15), it was speculated that treatment failure is likely caused by two factors: (i) a reinfection with a more resistant exogenous strain during treatment and (ii) resistance evolution of the primary strain (16–19). To distinguish between reinfection and resistance evolution, SNPs obtained by whole-genome sequencing (WGS) of strains were used to provide detailed information regarding drug resistance and its evolutionary events (20, 21). MTB infection is more genetically heterogeneous than traditionally thought (22). However, the microevolution and genetic resistance profiles acquired during treatment failure and identified on the molecular level by WGS have been even less studied.

We elucidated the current reality and the factors influencing treatment failure of MDR-TB in China and used WGS to further investigate the molecular evolution of strains during treatment failure, which has been less explored (19). These findings might reveal the current status of DR-TB treatment, which is crucial for improving future treatment strategies.

## RESULTS

### Basic information and DST profiles of baseline strains.

**FIG 1:**
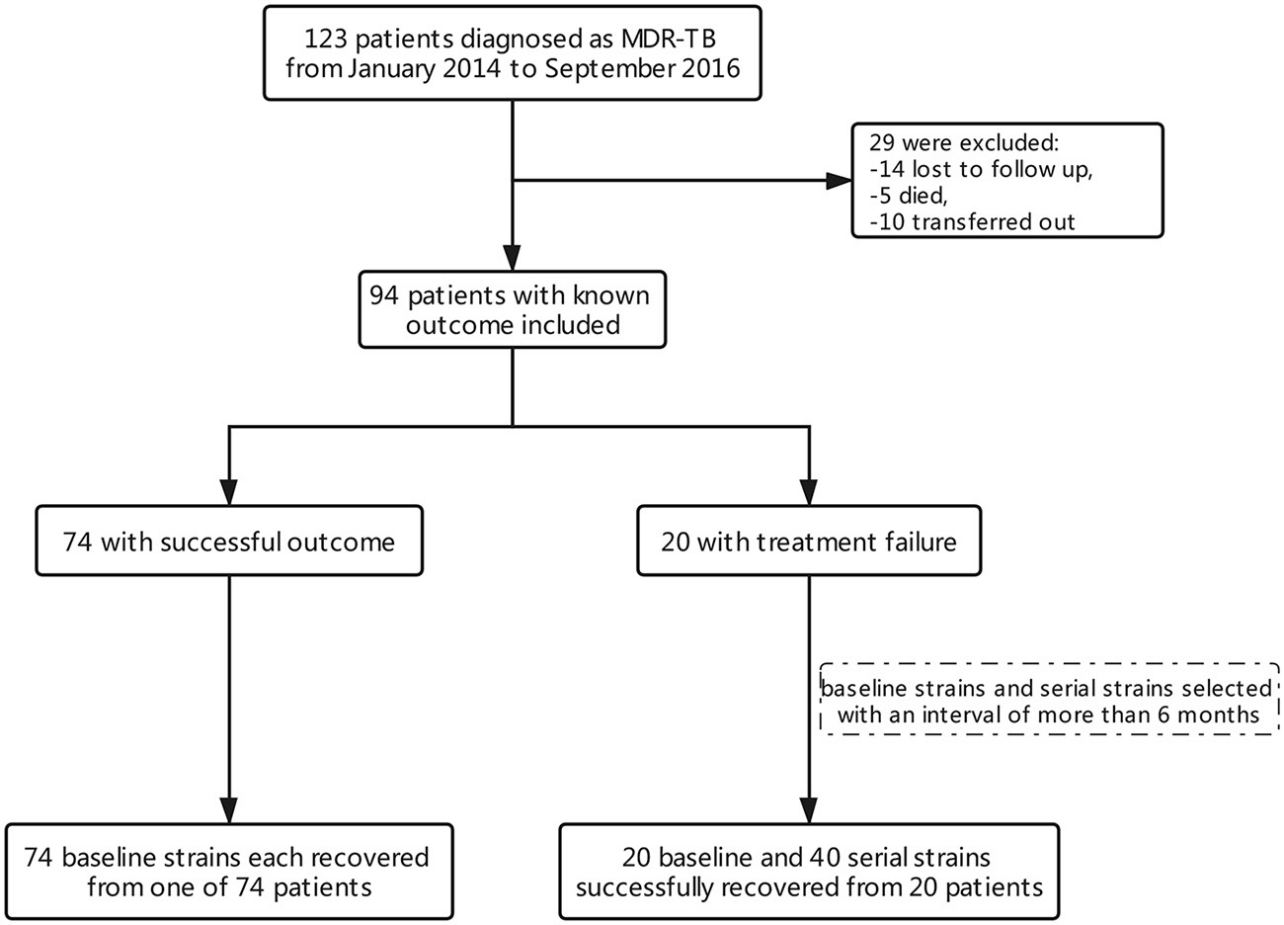
Flowchart of included and excluded patients and isolates in this study.

In total, 123 MDR-TB patients were diagnosed between January 2014 and September 2016. Among these, 94 patients had treatment outcomes by December 2018, of which 20 were treatment failure and 74 cured. We successfully revived 94 baseline strains from the 94 patients and 40 serial strains from the 20 treatment-failure patients (Fig. 1).

The characteristics of patients, such as history of TB treatment with or without second-line anti-TB drugs and previous treatment duration of over 1 year, were associated with poor treatment outcomes (Table S1 in the supplemental material).The higher resistance level of baseline strains (especially extensively drug resistant [XDR], which means resistant to both SLID [second-line injectable drug] and FQ [fluoroquinolone], or pre-XDR, which means resistant to either SLID or FQ, resistance to FQ or to pyrazinamide [PZA]) was also associated with poor treatment outcomes (Table 1).

**TABLE 1:** Treatment outcomes in relation to resistance patterns of baseline strains and administration of effective drugs

Parameter^*a*^	Successful outcomes (no. [%])	Poor outcomes (no. [%])	*P* value^*b*^	Risk ratio (95% CI) for treatment success^*b*^
Resistance Pattern of baseline strains			**<0.001**	**0.13 (0.04–0.45)**
MDR	40 (54.1)	2 (10.0)		
pre-XDR R to SLID	32 (43.2)	1 (5.0)		
pre-XDR R to FQ	1 (1.4)	15 (75.0)		
XDR	1 (1.4)	2 (10.0)		
Resistance to individual drugs				
EMB	41 (55.4)	15 (75.0)	0.113	0.84 (0.69–1.03)
PZA	34 (45.9)	17 (85.0)	**0.002**	**0.72 (0.58–0.89)**
LFX	33 (44.6)	17 (85.0)	**0.001**	**0.71 (0.57–0.88)**
MFX	11 (14.9)	10 (50.0)	**0.002**	**0.61 (0.40–0.92)**
SLID	2 (2.7)	3 (15.0)	0.107	0.49 (0.17–1.45)
SM	49 (66.2)	14 (70.0)	0.749	0.96 (0.78–1.20)
PTO	10 (13.5)	8 (40.0)	0.019	0.66 (0.43–1.01)
PAS	6 (8.1)	2 (10.0)	0.792	0.95 (0.63–1.44)
CS	3 (4.1)	6 (30.0)	0.002	0.40 (0.16–1.01)
No. of effective drugs in patient's treatment			**<0.001**	**0.04 (0.01–0.18)**
0–1	0 (0.0)	1 (5.0)		
2	2 (2.7)	6 (30.0)		
3	14 (18.9)	13 (65.0)		
≥4	58 (78.4)	0 (0.0)		
Effectiveness of specific drugs				
EMB	12 (16.2)	1 (5.0)	0.452	1.17 (0.97–1.41)
PZA	41 (55.4)	3 (15.0)	**<0.001**	**1.41 (1.14–1.75)**
FQ	25 (33.8)	5 (25.0)	**<0.001**	**1.68 (1.22–2.32)**
SLID	71 (96.0)	17 (85.0)	0.207	1.61 (0.72–3.62)
SM	0 (0)	0 (0.0)	/	/
PTO	62 (83.8)	9 (45.0)	**0.001**	**1.67 (1.12–2.50)**
PAS	10 (13.5)	4 (20.0)	0.712	0.89 (0.63–1.27)
CS	49 (66.2)	10 (50.0)	0.183	1.16 (0.92–1.48)
CFZ	4 (5.4)	5 (25.0)	0.027	0.54 (0.26–1.13)

a MDR, multidrug resistant; XDR, extensively drug resistant; SLID, second-line injectable drug; FQ, fluoroquinolone; EMB, ethambutol; PZA, pyrazinamide; LFX, levofloxacin; MFX, moxifloxacin; SM, streptomycin; PTO, prothionamide; PAS, para-aminosalicylic acid; CS, cycloserine; CFZ, clofazimine; /, not applicable.

b *P* value and risk ratio (95% CI) values given in boldface indicate statistical significance.

Importantly, insufficient administration of effective drugs was associated with poor clinical outcomes. In contrast, treatment regimens using specific effective drugs, such as PZA, levofloxacin (LFX), moxifloxacin (MFX), and prothionamide (PTO), were associated with successful outcomes (Table 1). We calculated the number of effective drugs by comparing the baseline DST and the initial treatment regimen. As the number of effective drugs decreased from ≥4, to 3, to ≤2 in the regimen of patients, treatment failure was observed to increase stepwise from 0% (0/58), to 48.1% (13/27), and to 77.8% (7/9), respectively (*P* < 0.001; relative risk [RR], 0.04, 95% confidence interval [CI] 0.01 to 0.18). Further analysis revealed no significant differences in the drug resistance profile or effective use of specific drugs in 27 patients treated with three effective drugs; however, any history of TB treatment with or without second-line anti-TB drugs was associated with treatment failure.

### Primary infection strain or reinfection distinguished by WGS.

**FIG 2:**
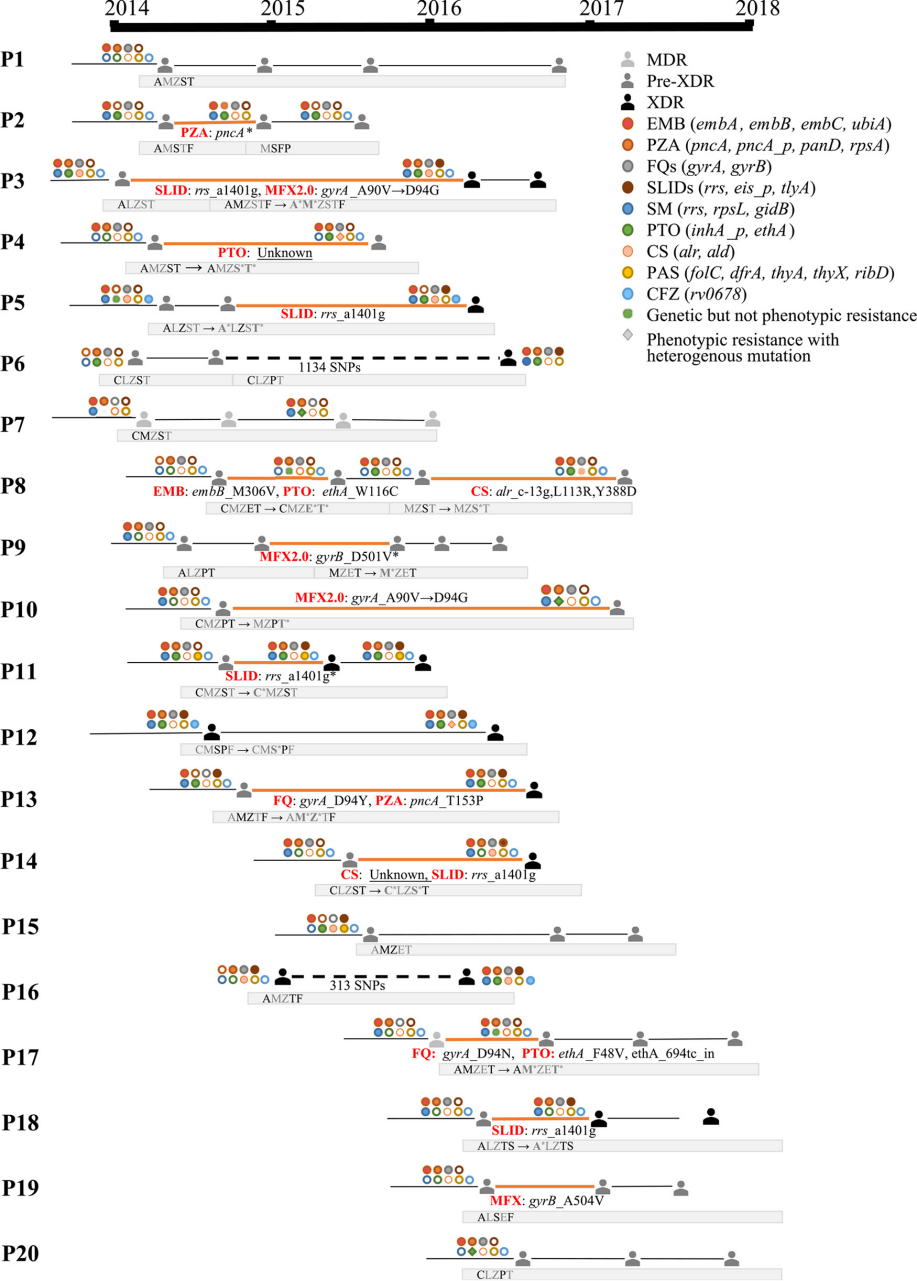
Evolution of drug resistance and treatment regimen for each patient. Different colors represent corresponding drugs. Solid circles represent resistance; hollow circle represents sensitivity. Gray lines under each patient show the treatment regimen and any changes to it. Letters in gray indicate ineffective use of this drug. Letters in bold with an asterisk indicate acquired resistance to this drug. E, ethambutol; Z, pyrazinamide; L, levofloxacin; M, moxifloxacin; A, amikacin; C, capreomycin; T, prothionamide; P, para-aminosalicylic acid; S, cycloserine; and F, clofazimine.

As reinfection by another higher-resistance MDR strain was considered a plausible reason for treatment failure, we used WGS to distinguish between primary infection and reinfection of 20 patients with poor treatment outcome. The number of different SNPs between the serial strains was in the range of 0 to 1,143. Eighteen (90.0%) cases were identified as the same strains, of which the number of SNP differences varied from 0 to 8 in this study. Only two cases (10%) were identified as reinfection by another, more resistant strain (no. 6 and no. 16), which had 1,134 and 313 SNPs in genetic distance, respectively (Fig. 2, Fig. S1 in the supplemental material).

Among the 18 cases infected with the same strain during the whole period of treatment, a total of 14 patients (77.8%) were observed to develop phenotypic or genotypic acquired drug resistance (ADR) (Table 2). The ADR events of FQ (both MFX 2.0 mg/ml and LFX) and second-line injectable drugs (SLID) were the most frequent, and were found in six (33.3%) and five (27.8%) out of 18 patients, respectively. The evolution from being drug susceptible to resistant was consistent in both the phenotype and genotype in most patients, except for the PTO phenotypic resistance of patient no. 4 and the cycloserine (CS) phenotypic resistance of patient no. 14, in which cases no mutations responsible for the acquired resistance were detected.

**TABLE 2:** Phenotypic or genotypic acquired resistance in patients of poor outcomes

Patient no.	Acquired phenotypic drug resistance^*a*^	Acquired genotypic drug resistance^*a*^	Genetic drug resistance consistent with phenotype^*b*^	Other mutations in drug resistance related genes^*b*^
1	/	/	/	/
2	PZA	PZA	*pncA*_L85R*, *pncA*_166c_in*	/
3	MFX2.0, SLID	FQ, SLID	*gyrA* A90V→D94N, *rrs*_a1401g	/
4	**PTO**	/	/	/
5	SLID	SLID	*rrs*_a1401g	*ethA* S57P*, *ethA* Y461stop*
7	/	/	/	*ethA* S183R*, *ethA* Q254P*, *ethA* Y286stop*
8	EMB, PTO, CS	EMB, PTO, CS	*embB* M306V; *ethA*_W116C*; *alr*_c-13g,L113R,Y388D*	*gyrB* T500A; *pncA* D8A*, *pncA* T142S*
9	MFX2.0	FQ	*gyrB*_E501D*	
10	MFX2.0	FQ	*gyrA*_A90V→D94G	/
11	SLID	SLID	*rrs*_a1401g*	/
12	/	/	/	/
13	FQ, PZA	FQ, PZA	*gyrA*_D94Y, *pncA*_T153P	/
14	SLID, **CS**	SLID	*rrs*_a1401g	/
15	**/**	**/**	/	/
17	FQ, PTO	FQ, PTO	*gyrA*_D94N; *ethA*_F48V*, *ethA*_694tc_in*	/
18	SLID	SLID	*rrs*_a1401g	/
19	MFX2.0	FQ	*gyrB*_A504V*	/
20	/	EMB, FQ	/	*embB*_Q497H, *gyrB*_D461N

a Boldface type indicates the genetic mutation responsible for resistance was not found. SLID, second-line injectable drug; FQ, fluoroquinolone; EMB, ethambutol; PZA, pyrazinamide; LFX, levofloxacin; MFX, moxifloxacin; PTO, prothionamide; CS, cycloserine; /, not applicable.

b *, hetero-resistance.

As no resistance-related mutation was discovered in the PTO-resistant isolate from patient no. 4 and the CS phenotypic resistance of patient no. 14, we further analyzed all differential SNPs identified between the serial isolates of these two patients. After excluding synonymous mutations, three mutations were identified to distinguish the resistant from the susceptible isolates of patient no. 14. These were Rv0565c Y306C (probable monooxygenase), Rne N649D (RNase E), and Rv2611c I102M (probable acyltransferase). Only two mutations were detected in the intergenic region of the isolate of patient no. 4, located in the promoter of *mprA* (mycobacterial persistence regulator protein) and *amount* genes (ammonium-transport integral membrane protein). These results provided a new insight into the acquired resistance of TB strains to drugs.

### Microevolution of resistance genes revealed by whole-genome sequencing.

Here, the evolution of resistance-related genes included the identification of the *pncA* mutation related to PZA (patients no. 2 and no. 13), the *gyrA* and *gyrB* mutations related to FQ (patients no. 3, no. 9, no. 10, no. 13, no. 17, and no. 19), the *rrs* mutation related to SLID (patients no. 3, no. 5, no. 11, no. 14, and no. 18), the *embB* mutation related to EMB (patients no. 8 and no. 20), the *alr* mutation related to CS (patients no. 8 and no. 14), and the *ethA* mutation related to PTO (patients no. 4, no. 8, and no. 17) (Table 2). Moreover, heterogeneous resistance, indicating the coexistence of either both drug-resistant and sensitive subpopulations or several drug-resistant subpopulations with different gene mutations, was found in 8 out of 14 patients. An ongoing change in the frequency of resistance mutations was detected by WGS in a series of isolates from nine patients (patients no. 2, 3, 5, 7, 8, 9, 11, 17, and 19), indicating the process of emergence or substitution of resistant subpopulations (Fig. 2, Table 3).

In patients no. 8, no. 11, and no. 19, the emergence, selection, and fixation process of a drug resistance-related mutation were entirely recorded by WGS. In detail, the frequency of *ethA* W116C of patient no. 8, *rrs* a1401g of patient no. 11, and *gyrB* A504V of patient no. 19 increased from 0% at the initiation of treatment to nearly 100% in the last cultured isolates.

Interestingly, we found a mutation shift from a low-level resistance mutation to a different high-level resistance mutation. In patient no. 3, the *gyrA* D94N mutation, usually characterized by high level of resistance to FQ, gradually replaced the existing A90V mutation, resulting in low level of resistance to FQ. This phenomenon was also observed in isolates from patient no. 10, in which the *gyrA* D94G mutation was demonstrated to have replaced the previous A90V mutation during treatment containing MFX (Table 2, Table 3).

**TABLE 3:** Mutation frequencies changing gradually for resistance-related mutations as detected by WGS

Patient no.	Acquired phenotypic drug resistance^*a*^	Acquired genetic drug resistance	Resistant mutations	Frequency of resistant mutations
2	PZA	*pncA*	L85R	0 ↗ 15.5 ↘ 0
			166c in	0 ↗ 35.7 ↘ 0
3	MFX2.0	*gyrA*	A90V	98.2 ↘ 83.5 ↘ 0
			D94N	0 ↗ 81.7 ↗ 84.6
5	/	*ethA*	S57P	54.9 ↘ 0 ↗ 99.6
			Y461stop	34.9 ↗ 99.2 ↘ 0
7	/	*ethA*	S183R	42.1 ↘ 15.1 ↘ 0 → 0
			Q254P	0 ↗ 59.9 ↘ 0 → 0
			Y286stop	34.3 ↘ 23.6 ↗ 99.8 → 99.8
8	CS	*alr*	c-13g	0 → 0 → 0 ↗ 83.6
			L1134	0 → 0 → 0 ↗ 15.0
			Y388D	0 → 0 → 0 ↗ 44.4
	PTO	*ethA*	W116C	0 ↗ 29.6 ↗ 98.2 → 98.2
9	MFX2.0	*gyrA*	D94H	0 → 0 → 22.8 ↘ 0 → 0
		*gyrB*	E501V	0 → 0 ↗ 24.3 ↗ 47.1 ↘ 29.6
			E501D	0 → 0 → 0 ↗ 48.7 ↗ 51.3
11	SLID	*rrs*	a1401g	0 ↗ 79.5 ↗ 99.7
17	PTO	*ethA*	F48V	0 ↗ 88.7 ↘ 62.2 ↘ 20.9
			694tc_in	0 ↗ 10.9 ↗ 28.2 ↗ 82.4
19	MFX2.0	*gyrB*	A504V	0 ↗ 24.8 ↗ 86.0

a SLID, second-line injectable drug; PZA, pyrazinamide; MFX, moxifloxacin; PTO, prothionamide; CS, cycloserine; /, not applicable.

In patients no. 5, 7, 9, and 17, several different drug-resistant subpopulations were coexisting. In patient no. 5, two mutations in the *ethA* gene were found at the start of treatment, *ethA* S57P and *ethA* Y461*, with frequencies of 54.9% and 34.9%, respectively. In the second (6 months later) and third (23 months later) specimens, only the *ethA* S57P or *ethA* Y461* mutations were present (Table 3). Similarly, in patient 7, three different subpopulations with drug resistance mutations of *ethA* were gradually fixed to carry *ethA* Y286* as the only mutation during ineffective treatment with PTO. The strain of patient no. 17 was initially sensitive to PTO, exhibiting a wild-type *ethA*. After 6 months of ineffective treatment, *ethA* F48V and *ethA* 694t-insertion mutations were both detected with frequencies of 88.7% and 10.9%, respectively. After another 16 months of continued treatment, the subpopulation carrying the *ethA* 694t-insertion became the majority, with its proportion rising to 82.4%, whereas the proportion of the subpopulation carrying the *ethA* F48V mutation decreased to 20.9%.

## DISCUSSION

The WGS method is widely used in distinguishing relapse from exogenous reinfection in cases of recurrence after treatment completion, revealing that about 60% are relapse and 40% are reinfection (10, 20, 23–25). However, few studies have investigated this issue in MDR-TB treatment failure patients, who were hard to treat and remained a source of infection during unsuccessful treatment. The majority of paired strains (18/20, 90.0%) persisted genetically unchanged (SNPs ≤8), consistent with previous studies by WGS (21, 26), but higher than previous studies in which the genotype was defined by mycobacterial interspersed repetitive units-variable number of tandem repeats (MIRU-VNTR) (18, 27, 28). The reason for the lower frequency of reinfection could be that small changes in MIRU-VNTR can make closely related strains appear very different, but the higher resolution WGS method can distinguish that these strains are actually very similar. Since the study was conducted in Wenzhou City, Zhejiang Province, there could be fewer transmission incidents in this city than in the communities sampled in other studies (21, 29).

Deep analysis of the failure cases might lead to a road of better treatment options for MDR-TB. Here, administration of an insufficient number of effective drugs was strongly associated with treatment failure and acquired drug resistance events, strengthening the previous results defining the genotype by MIRU-VNTR (13). We performed a comprehensive and standardized DST for all second-line antituberculosis drugs available at that time, remedying the limitation that previous studies did not include DST results for PZA, MFX, and PTO. Another strength of this study was that WGS-based mutations were also analyzed to confirm the acquired phenotypic resistance, highlighting the importance of DST in the treatment of DR-TB (17, 30).

In total, among patients persistently infected with the same strain, 77.8% patients (14/18) were noted to develop acquired drug resistance, which was higher than that reported previously. A previous study only performed DST of FQ and SLID at the initiation and checkpoint of treatment to determine the resistance pattern, which might have underestimated the prevalence of acquired resistance to other drugs (13, 28). The acquired resistance to FQ is the most common evolutionary event in the duration of MDR-TB treatment (13). However, in this study, as 83.8% (15/18) patients were initially resistant to FQ (resistance to either LFX or MFX), we did not focus much on the evolution event of the resistance to FQ. Acquired resistance to SLID and higher resistance level to MFX (from 0.5 mg/ml to 2.0 mg/ml) was the most frequent event and detected in five and four patients, respectively, followed by the acquired resistance to LFX (from sensitivity to resistance), PZA, PTO, CS, and ethambutol (EMB) found in two patients.

Our study reemphasized the importance of the usage of FQ in treatment (31), as the success rate of treatment was significantly higher in FQ-S patients effectively treated with FQ, in accordance with previously reported conclusions (32, 33). The usage of MFX to treat LFX-resistant MDR-TB patients, regardless of the effectiveness of the treatment combination, resulted in the evolution of a higher level of resistance to FQ (34), a finding that merits serious attention. In patients no. 3 and no. 10, the originally identified *gyrA* A90V resistance mutation (low level) was replaced by mutations with higher levels of resistance, *gyrA* D94N and D94G (35). Accordingly, in patients no. 9 and no. 19, carrying the *gyrA* A90V low-level resistance mutation, the emergence of *gyrB* mutations resulted in higher level of resistance to MFX.

Heterogeneous drug resistance is a common reason for discordant results in phenotypic and genotypic DSTs. The coexistence of subpopulations with different drug resistance profiles in the same patient might result in unfavorable responses to treatment, and might lead to treatment failure (36–39). Accordingly, we noted the simultaneous coexistence of multiple subpopulations containing different drug-resistant mutations in the early isolates of patients no. 3, 5, 7, and 17. After a period of drug pressure screening, a single subpopulation began to dominate, and in some cases became the sole population, with the mutation reaching 100% frequency, suggesting that the selection of TB resistance is common under long-term infection and drug pressure (39–42). Hence, these highly resistant and relatively fit TB bacteria might be involved in the spread from the early steps of infection (43). These findings underscore the importance of early detection of resistance, especially the detection of the resistant subpopulations before and during the treatment of DR-TB. As a highly sensitive molecular detection method, WGS could be used to diagnose these drug-resistant subgroups early, thereby reducing the possibility of being screened as fully drug-resistant infections in the future.

Most cases of phenotypic drug resistance can be explained by mutations in drug resistance-related genes. However, no corresponding molecular resistance mutations were found in two of these strains, resistance to PTO in patient no. 4 and resistance to CS in patient no. 14. We targeted a few SNPs during the microevolution of these resistance cases, which requires subsequent *in vitro* validation and screening in a larger population.

This study had the following limitations. First, the treatment regimen was based on the 2011 version of the WHO guidelines, what was available and used at that time. The order of recommendation for the administration of some of the drugs has since changed. As a retrospective study, DSTs could not be provided to the clinic in a timely manner. Second, any compliance issues of patients and loss of follow-up were ignored. Third, due to the procedure of revival of sputum specimens and the decontamination operations involved in this process, some subpopulations might have been lost and become undetected in WGS analysis. Third, as this was a single-center study, it included an insufficient number of MDR-TB patients. Finally, other factors that might influence treatment outcome, such as host-pathogen interaction or socioeconomic status, were not investigated in this study.

In this study, the main reason for the treatment failure of MDR-TB patients was insufficient effective drugs, which may lead to higher levels of drug resistance in MDR-TB strains. Therefore, the study emphasizes the importance of DST of second-line drugs when implementing the second-line drug regimen in MDR-TB patients. WGS detects low-frequency resistance mutations and emerging heterogeneous resistance with high sensitivity, which is of great significance for guiding clinical treatment and preventing acquired resistance.

## MATERIALS AND METHODS

### Clinical MDR-TB cases.

We retrospectively included all MDR-TB patients from January 2014 to September 2016 in the Wenzhou Central Hospital (Zhejiang Province, China) with a known outcome according to the standard outcome definitions by WHO (3). DST of first-line antituberculosis agents (isoniazid [INH] and rifampin [RIF]) was conducted routinely on positive cultures by MGIT 960 to determine whether it was MDR. Patients were divided into two groups according to the treatment outcome: treatment success and treatment failure. The treatment-success group included patients who had been either cured or had completed treatment. The treatment-failure group included patients exhibiting lack of conversion by the end of the intensive phase or bacteriological reversion in the continuation phase after conversion to negative. As these patients received treatment from 2014 to 2016, the WHO guidelines for the programmatic management of the drug-resistant tuberculosis 2011 update (14) was the only one available and used at that time. This standardized treatment combination included the administration of pyrazinamide (PZA), a second-line injectable drug (SLID) (amikacin [AM], kanamycin [KM], or capreomycin [CM]), a fluoroquinolone (levofloxacin [LFX], or moxifloxacin [MFX]), and two of cycloserine (CS), prothionamide (PTO), *p*-aminosalicylic acid (PAS), clofazimine (CFZ), or ethambutol (EMB) for the period of 6 to 8 months during the intense phase, continued with administration of a combination of 3 to 4 drugs without a SLID for another 18 months.

Clinical information, including age, gender, comorbid conditions, treatment history, and treatment regimen was extracted from the medical records of patients to analyze the risk factors related to treatment outcomes. Baseline strains, the first sputum culture positive strain, of all enrolled patients were collected and revived from refrigeration. For all treatment failure patients, sputum specimens were collected every month for the first 6 months, and then every 1 to 2 months according to WHO guidelines. In order to observe evolution events and heterogeneity, we selected at least two more serial strains at intervals of more than 6 months. Strains that failed to revive or were contaminated were excluded. All colonies on the plate were scraped off and resuspended in 30% glycerol before freezing.

The study was approved by the Ethics Committee of Wenzhou Central Hospital, Zhejiang Province, China. All patients provided written informed consent before the study.

### Phenotypic drug susceptibility tests.

Drug susceptibility tests (DSTs) were conducted on all included strains to compare the resistance profiles between the two groups. Following the revival of strains on Löwenstein–Jensen medium, we performed DSTs for 14 drugs (44–50) on the baseline strains of all patients and the series strains of treatment failure patients, namely, INH (0.2 μg ml^−1^), RIF (40.0 μg ml^−1^), EMB (2.0 μg ml^−1^), LFX (2.0 μg ml^−1^), MFX (2.0 μg ml^−1^), AM (30.0 μg ml^−1^), KM (30.0 μg ml^−1^), CM (40.0 μg ml^−1^), streptomycin (SM) (4.0 μg ml^−1^), PAS (1.0 μg ml^−1^), CFZ (1.0 μg ml^−1^), and CS (30.0 μg ml^−1^) using the proportion method on Löwenstein–Jensen medium (Baso, Zhuhai, Guangzhou Province, China). Exceptions were for PZA (100.0 μg ml^−1^) and PTO (2.5 μg ml^−1^), the DSTs of which were performed using an automated MGIT 960 (Becton, Dickinson Diagnostic Systems, Franklin Lakes, NJ, USA) according to the manufacturer's guidelines. We conducted the DSTs of EMB, PZA, and PTO at least twice due to their unsatisfactory reproducibility. Where the results were inconsistent, a third test was performed and the interpretation was based on the two most consistent results out of the total three.

### Whole-genome sequencing.

Whole-genome sequencing (WGS) was performed on all selected strains to provide genotypic resistance, distinguish between a continuous infection with the primary strain or reinfection, and elucidate the microevolution of serial strains. Growth was scraped from Löwenstein–Jensen medium. Genomic DNA was extracted using the Qiagen DNeasy blood and tissue kit (Cat. no. 69506) and sequencing libraries were constructed using the Nextera XT sample prep kit (Illumina, San Diego, CA, USA) following the manufacturer's protocol. All strains were sequenced on an Illumina Miseq or X 10 with at least 100-fold coverage.

WGS data were analyzed (51) according to previous studies. After filtering the low-quality reads, the reads were aligned to the MTB H37Rv (GenBank NC_000962.3) reference sequence using Bowtie2 (version 2.3.3.1) with default parameters. Single nucleotide polymorphisms (SNPs) were detected using SAMtools (version 1.6) with a minimum sequencing depth of 10 reads without strand bias and a frequency of no less than 10%. All SNPs with a mutation frequency exceeding 10% were retained in the step of calling SNPs to detect microevolution and minor subpopulations. If the mutation ratio of a certain site was above 80%, it was considered a fixed mutation.

SNPs in highly repetitive regions such as PE and PPE, GC-enriched sequences, and drug resistance-related genes were removed from the analysis. The phylogenetic tree was constructed using the maximum likelihood method using MEGA (version 7.0) software based on the fixed SNPs, with H37Rv as the root. The strains with a genetic distance of no more than 12 SNPs were considered persistent infection with the same strain.

### Ethical approval.

The study was approved by the Ethics Committee of Wenzhou Central Hospital, Zhejiang Province, China. All patients provided written informed consent before the study.

### Statistical analysis.

We used the chi-square test or Fisher's exact test to calculate significant differences between characteristics of patients, drug resistance, and treatment regimen and outcome. We performed univariable logistic regression analysis and calculated the relative risk (RR) followed by its 95% confidence intervals. Variables with a *P* < 0.05 were considered significant. We performed all analyses using the SPSS version 21 software.

### Data availability.

The raw data of WGS are submitted to the SRA database and can be accessed by the public via BioProject accession number PRJNA613359. The SRA accession numbers for these isolates are SAMN14402132 to SAMN14402172. The WGS data for a portion of these strains were previously made available to the public under BioProject number PRJNA522942 (51).

10.1128/mSphere.00884-20.1 TABLE S1 Treatment outcomes of MDR-TB patients according to patient characteristics; *P* value and risk ratio (95% CI) values given in boldface indicate statistical significance. Download Table S1, DOCX file, 0.01 MB. Copyright © 2020 Chen et al. 2020 Chen et al. This content is distributed under the terms of the Creative Commons Attribution 4.0 International license.

10.1128/mSphere.00884-20.2 TABLE S2 Diagnostic criteria of genetic sequencing for genotypic resistance. Download Table S2, DOCX file, 0.02 MB. Copyright © 2020 Chen et al. 2020 Chen et al. This content is distributed under the terms of the Creative Commons Attribution 4.0 International license.

10.1128/mSphere.00884-20.3 FIG S1 Phylogenetic tree of 60 serial strains in 20 patients with treatment failure; branch in red means reinfection by different strains during treatment. Download FIG S1, DOCX file, 0.1 MB. Copyright © 2020 Chen et al. 2020 Chen et al. This content is distributed under the terms of the Creative Commons Attribution 4.0 International license.
