# Supplementary material for: Analysis of Serial Multidrug-Resistant Tuberculosis Strains Causing Treatment Failure and Within-Host Evolution by Whole-Genome Sequencing
Source: mSphere. 2020 Dec 23;5(6):e00884-20. doi: 10.1128/mSphere.00884-20 (PMC7763549; doi:10.1128/mSphere.00884-20)
Supplement: TABLE S1 [file mSphere.00884-20-st001.docx]

|  | Successful Outcomes, No.(%) | Poor Outcomes, No.(%) | P Value | Risk Ratio (95% CI) for Treatment Success |
| --- | --- | --- | --- | --- |
| **Patient Characteristics** |  |  |  |  |
| Sex |  |  | 0.849 | 0.98 |
| Female | 17 (23.0) | 5 (25.0) |  | (0.76-1.26) |
| Male | 57 (77.0) | 15 (75.0) |  |  |
| Quartile of age, y |  |  | 0.777 | 0.94 |
| <60 | 69 (93.2) | 19 (95.0) |  | (0.65-1.37) |
| ≥60 | 5 (6.8) | 1 (5.0) |  |  |
| Diabetes mellitus |  |  | 0.452 | 0.92 |
| Yes | 23 (31.1) | 8 (40.0) |  | (0.72-1.17) |
| No | 51 (68.9) | 12 (60.0) |  |  |
| previous treatment history |  |  | **0.011** | **0.75** |
| Yes | 49 (66.2) | 19 (95.0) |  | **(0.63-0.89)** |
| No | 25 (33.8) | 1 (5.0) |  |  |
| previous treatment history with SLDs |  |  | **0.000** | **0.62** |
| Yes | 18 (24.3) | 14 (70.0) |  | **(0.45-0.85)** |
| No | 56 (75.7) | 6 (30.0) |  |  |
| previous treatment duration, y |  |  | **0.016** | **1.29** |
| ≤1 | 37 (50.0) | 4 (20.0) |  | **(1.06-1.59)** |
| >1 | 37 (50.0) | 16 (80.0) |  |  |
| Caviraty on chest radiograph |  |  | 0.394 | 0.86 |
| Yes | 58 (78.4) | 18 (90.0) |  | (0.70-1.06) |
| No | 16 (21.6) | 2 (10.0) |  |  |
| Extrapulmonary tuberculosis disease |  |  | 0.107 | 0.49 |
| Yes | 2 (2.7) | 3 (15.0) |  | (0.17-1.45) |
| No | 72 (97.3) | 17 (85.0) |  |  |
| Lineage |  |  | 0.818 | 1.296 |
| Lineage 2 | 55 (74.3) | 16 (80.0) |  | (0.48-3.49) |
| Lineage 4 | 19 (25.7) | 4 (20.0) |  |  |
