# Supplementary material for: Analysis of Serial Multidrug-Resistant Tuberculosis Strains Causing Treatment Failure and Within-Host Evolution by Whole-Genome Sequencing
Source: mSphere. 2020 Dec 23;5(6):e00884-20. doi: 10.1128/mSphere.00884-20 (PMC7763549; doi:10.1128/mSphere.00884-20)
Supplement: TABLE S2 [file mSphere.00884-20-st002.docx]

| Drugs | Resistance –related genes | Resistance-related mutations | added mutations in this study |
| --- | --- | --- | --- |
| INH^[1-4]^ | *katG* | W90R, R104Q, A109V, G125D, L141F, D142G, L159P, T180K, G182R, W191G/R, P232R, G297V, W300C, S315I/N/T, W328L, S481L, A614E, V633A, S700P, L704S, pooled frameshifts and premature stop codons | Q88P+M257V, W91R, A122D, Q127P, A312E, D419Y |
|  | *inhA* | t-8c, c-15t, g-17t, I21T, S94A, I194T | / |
|  | *ahpC* | g-48a, c-57t, c-72t | c-52t |
|  | *nat* | Y188H |  |
|  | *ndh* | R13C, T110A, R268H |  |
|  | *iniA* | P3A, R537H |  |
|  | *iniB* | Pooled framshift |  |
|  | *iniC* | t79ins, a98ins, W83G |  |
| RIF^[1-3, 5]^ | *rpoB* | V170F, V359A, L430P, S431T, Q432K/L/P, M434I, D435A/F/G/N/V/Y, S441L/Q, H445C/D/F/G/L/N/P/R/Y, S450F/L/Q/W/Y, L452P, I491F, D545E, T676P, G981D | S450V, S450P, S450H, H445Q+L452S, 1291_gcc_in |
| EMB^[1-3, 5-12]^ | *embB* | M306I/V, D328Y, D354A, G406A/D/S/C, Q497K/R/P, N1033K | / |
|  | *embA* | c-12t, c-16g, c-16t | / |
|  | *embC* | T270I, D329G, N394D | / |
|  | *embR* | P49A+P243S | / |
|  | *ubiA* | L31P, A35E/S, A38V, V55G/M, V148A, G165C, S173A, K174R, W175G, F176L, I179T, M180V, V188A, V229G, L235P, A237V, R240C, S244T, A249G, A278V | / |
| PZA^[2, 3, 13-18]^ | *pncA* | any amino acid change, pooled frameshifts and premature stop codons | / |
|  | *panD* | M117T, E126*, A128S, E130G, P134S, L136R, V138A/G/E, M171I | / |
|  | *rpsA* | / | / |
| FQ^[2]^ |  |  |  |
| LFX | *gyrA* | G88A, G88C, S91P, A90V, D94A, D94G, D94H, D94N, D94Y | / |
|  | *gyrB* | E459K, A504V | / |
| MFX | *gyrA* | G88C, A90V, S91P, D94A, D94G, D94N, D94Y | / |
| SM^[2, 3, 19]^ | *rpsL* | K43R, K43T, K88Q, K88R, T40I | / |
|  | *rrs* | a514c, a514t, c462t, c513t, c517t |  |
|  | *gidB* | / | Pooled framshifts |
| SLI^[2, 3]^ |  |  |  |
| AM | *rrs* | a1401g, g1484t | / |
| KM | *eis* | c-14t, g-10a | / |
|  | *rrs* | a1401g, c1402t, g1484t | / |
| CM | *rrs* | a1401g, c1402t, g1484t | / |
|  | *tlyA* | N236K, pooled frameshifts and premature stop codons | / |
| PTO^[2, 4, 20]^ | *inhA* | c-15t, c-15t+I194T, c-15t+S49A | / |
|  | *ethA* | / | pooled frameshifts and premature stop codons |
| PAS^[21-24]^ | *folC* | / | E40G/K/Q, I43A/F/S/T/V, R49P/W, L56V, N73S, R91W D111A, G112S, D135A, S150G/C/R, F152S/L,E153A/G, V256A, S335I, R410W, A420V, E434Q, A457V |
|  | *thyA* | / | G15R, T22I, Y36C, H75N, G76*, V77F, W83C/*, G91E/R, W98*, S105P, R126Q, F152V, C161T, L183V, R127L, N134K, L143P, L146R, H147N, L172P, A182P, Q191R, H207R, I211V, P224L, R235P, 1A259P, V261G, V263I/G, R264* |
|  | *dfrA* | / | / |
|  | *ribD* | / | g-11a, G8R |
| CLO^[25-29]^ | *rv0678* | / | any amino acid change, pooled frameshifts and premature stop codons |
|  | *rv1979c* | V351A | / |
|  | *rv2535c (pepQ)* | pooled frameshifts and premature stop codons | / |
|  | *mmpL3* | / | / |
|  | *mmpL5* | / | / |

References:

[1] ALLIX-BEGUEC C, ARANDJELOVIC I, BI L, et al. Prediction of Susceptibility to First-Line Tuberculosis Drugs by DNA Sequencing[J]. N Engl J Med, 2018,379(15):1403-1415.

[2] MIOTTO P, TESSEMA B, TAGLIANI E, et al. A standardised method for interpreting the association between mutations and phenotypic drug resistance in Mycobacterium tuberculosis[J]. European Respiratory Journal, 2017,50(6):1701354.

[3] WALKER T M, KOHL T A, OMAR S V, et al. Whole-genome sequencing for prediction of Mycobacterium tuberculosis drug susceptibility and resistance: a retrospective cohort study[J]. Lancet Infect Dis, 2015,15(10):1193-1202.

[4] VILCHEZE C, JACOBS W J. Resistance to Isoniazid and Ethionamide in Mycobacterium tuberculosis: Genes, Mutations, and Causalities[J]. Microbiol Spectr, 2014,2(4):M2-M14.

[5] FARHAT M R, SULTANA R, IARTCHOUK O, et al. Genetic Determinants of Drug Resistance in Mycobacterium tuberculosis and Their Diagnostic Value[J]. American Journal of Respiratory and Critical Care Medicine, 2016,194(5):621-630.

[6] GIRI A, GUPTA S, SAFI H, et al. Polymorphisms in Rv3806c ( ubiA ) and the upstream region of embA in relation to ethambutol resistance in clinical isolates of Mycobacterium tuberculosis from North India[J]. Tuberculosis, 2018,108:41-46.

[7] HE L, WANG X, CUI P, et al. ubiA (Rv3806c) encoding DPPR synthase involved in cell wall synthesis is associated with ethambutol resistance in Mycobacterium tuberculosis[J]. Tuberculosis (Edinb), 2015,95(2):149-154.

[8] LINGARAJU S, RIGOUTS L, GUPTA A, et al. Geographic Differences in the Contribution of ubiA Mutations to High-Level Ethambutol Resistance in Mycobacterium tuberculosis[J]. Antimicrobial Agents and Chemotherapy, 2016,60(7):4101-4105.

[9] TULYAPRAWAT O, CHAIPRASERT A, CHONGTRAKOOL P, et al. Association of ubiA mutations and high-level of ethambutol resistance among Mycobacterium tuberculosis Thai clinical isolates[J]. Tuberculosis (Edinb), 2019,114:42-46.

[10] XU Y, JIA H, HUANG H, et al. Mutations Found in embCAB, embR, and ubiA Genes of Ethambutol-Sensitive and -Resistant Mycobacterium tuberculosis Clinical Isolates from China[J]. Biomed Res Int, 2015,2015:951706.

[11] KHOSRAVI A D, SIROUS M, ABDI M, et al. Characterization of the most common embCAB gene mutations associated with ethambutol resistance in Mycobacterium tuberculosis isolates from Iran[J]. Infect Drug Resist, 2019,12:579-584.

[12] SUN Q, XIAO T, LIU H, et al. Mutations within embCAB Are Associated with Variable Level of Ethambutol Resistance in Mycobacterium tuberculosis Isolates from China[J]. Antimicrobial Agents and Chemotherapy, 2018,62(1).

[13] PANKHURST L J, DEL O E C, VOTINTSEVA A A, et al. Rapid, comprehensive, and affordable mycobacterial diagnosis with whole-genome sequencing: a prospective study[J]. Lancet Respir Med, 2016,4(1):49-58.

[14] ZIGNOL M, CABIBBE A M, DEAN A S, et al. Genetic sequencing for surveillance of drug resistance in tuberculosis in highly endemic countries: a multi-country population-based surveillance study[J]. The Lancet Infectious Diseases, 2018.

[15] YADON A N, MAHARAJ K, ADAMSON J H, et al. A comprehensive characterization of PncA polymorphisms that confer resistance to pyrazinamide[J]. Nat Commun, 2017,8(1):588.

[16] SHI W, CHEN J, FENG J, et al. Aspartate decarboxylase (PanD) as a new target of pyrazinamide in Mycobacterium tuberculosis[J]. Emerg Microbes Infect, 2014,3(8):e58.

[17] ZHANG S, CHEN J, SHI W, et al. Mutations in panD encoding aspartate decarboxylase are associated with pyrazinamide resistance in Mycobacterium tuberculosis[J]. Emerg Microbes Infect, 2013,2(6):e34.

[18] GU Y, YU X, JIANG G, et al. Pyrazinamide resistance among multidrug-resistant tuberculosis clinical isolates in a national referral center of China and its correlations with pncA, rpsA, and panD gene mutations[J]. Diagn Microbiol Infect Dis, 2016,84(3):207-211.

[19] WONG S Y, LEE J S, KWAK H K, et al. Mutations in gidB confer low-level streptomycin resistance in Mycobacterium tuberculosis[J]. Antimicrob Agents Chemother, 2011,55(6):2515-2522.

[20] BROSSIER F, VEZIRIS N, TRUFFOT-PERNOT C, et al. Molecular investigation of resistance to the antituberculous drug ethionamide in multidrug-resistant clinical isolates of Mycobacterium tuberculosis[J]. Antimicrob Agents Chemother, 2011,55(1):355-360.

[21] CHENG V W, LEUNG K S, KWOK J S, et al. Phylogenetic and Structural Significance of Dihydrofolate Synthase (folC) Mutations in Drug-Resistant Mycobacterium tuberculosis[J]. Microb Drug Resist, 2016,22(7):545-551.

[22] ZHANG X, LIU L, ZHANG Y, et al. Genetic determinants involved in p-aminosalicylic acid resistance in clinical isolates from tuberculosis patients in northern China from 2006 to 2012[J]. Antimicrob Agents Chemother, 2015,59(2):1320-1324.

[23] ZHAO F, WANG X D, ERBER L N, et al. Binding pocket alterations in dihydrofolate synthase confer resistance to para-aminosalicylic acid in clinical isolates of Mycobacterium tuberculosis[J]. Antimicrob Agents Chemother, 2014,58(3):1479-1487.

[24] MATHYS V, WINTJENS R, LEFEVRE P, et al. Molecular genetics of para-aminosalicylic acid resistance in clinical isolates and spontaneous mutants of Mycobacterium tuberculosis[J]. Antimicrob Agents Chemother, 2009,53(5):2100-2109.

[25] ISMAIL N, OMAR S V, ISMAIL N A, et al. Collated data of mutation frequencies and associated genetic variants of bedaquiline, clofazimine and linezolid resistance in Mycobacterium tuberculosis[J]. Data Brief, 2018,20:1975-1983.

[26] HARTKOORN R C, UPLEKAR S, COLE S T. Cross-Resistance between Clofazimine and Bedaquiline through Upregulation of MmpL5 in Mycobacterium tuberculosis[J]. Antimicrobial Agents and Chemotherapy, 2014,58(5):2979-2981.

[27] ZHANG S, CHEN J, CUI P, et al. Identification of novel mutations associated with clofazimine resistance in Mycobacterium tuberculosis[J]. J Antimicrob Chemother, 2015,70(9):2507-2510.

[28] ALMEIDA D, IOERGER T, TYAGI S, et al. Mutations in pepQ Confer Low-Level Resistance to Bedaquiline and Clofazimine in Mycobacterium tuberculosis[J]. Antimicrob Agents Chemother, 2016,60(8):4590-4599.

[29] LI W, SANCHEZ-HIDALGO A, JONES V, et al. Synergistic Interactions of MmpL3 Inhibitors with Antitubercular Compounds In Vitro[J]. Antimicrob Agents Chemother, 2017,61(4).
