# Supplementary figures and images for: Analysis of Serial Multidrug-Resistant Tuberculosis Strains Causing Treatment Failure and Within-Host Evolution by Whole-Genome Sequencing
Source: mSphere. 2020 Dec 23;5(6):e00884-20. doi: 10.1128/mSphere.00884-20 (PMC7763549; doi:10.1128/mSphere.00884-20)

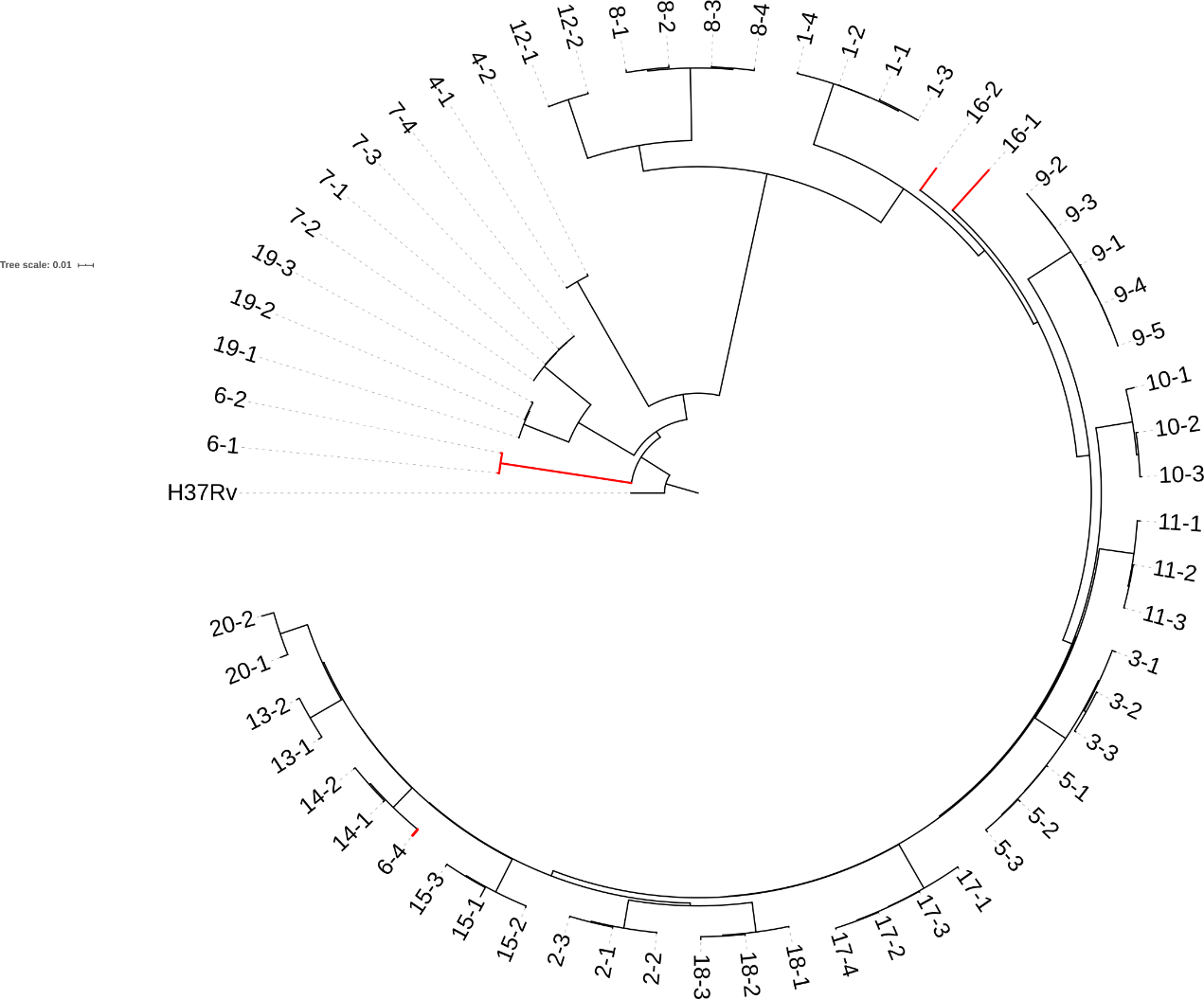

Supplement: FIG S1 [file mSphere.00884-20-sf001.docx]
